# Effect of Surface-Modified TiO_2_ Nanoparticles on the Anti-Ultraviolet Aging Performance of Foamed Wheat Straw Fiber/Polypropylene Composites

**DOI:** 10.3390/ma10050456

**Published:** 2017-04-26

**Authors:** Lihui Xuan, Guangping Han, Dong Wang, Wanli Cheng, Xun Gao, Feng Chen, Qingde Li

**Affiliations:** 1 Key Laboratory of Bio-based Material Science and Technology (Ministry of Education), Northeast Forestry University, Harbin 150040, China; leeh91@hotmail.com (L.X.); guangping.han@nefu.edu.cn (G.H.); zcwangd@hotmail.com (D.W.); gaoxunbeihuauniversity@hotmail.com (X.G.); c.f84@hotmail.com (F.C.); liqingde2017@hotmail.com (Q.L.); 2 Wood Engineering Department, Liaoning Forestry Vocational-Technical College, Shenyang 110101, China; 3 College of Arts and Design, Qiqihar University, Qiqihar 161000, China

**Keywords:** surface-modified TiO_2_ nanoparticles, foamed wheat straw fiber/polypropylene composites, mechanical properties, thermostability, UV stability

## Abstract

Surface modification and characterization of titanium dioxide (TiO_2_) nanoparticles and their roles in thermal, mechanical, and accelerated aging behavior of foamed wheat straw fiber/polypropylene (PP) composites are investigated. To improve the dispersion of nanoparticles and increase the possible interactions between wheat straw fiber and the PP matrix, the surface of the TiO_2_ nanoparticles was modified with ethenyltrimethoxy silane (A171), a silane coupling agent. The grafting of A171 on the TiO_2_ nanoparticles’ surface was characterized by Fourier transform infrared spectroscopy (FTIR). The wheat straw fibers treated with A171 and modified TiO_2_ nanoparticles were characterized by FTIR and thermogravimetric analysis (TGA). FTIR spectra confirmed that the organic functional groups of A171 were successfully grafted onto the TiO_2_ nanoparticles and wheat straw fibers, and the modified TiO_2_ nanoparticles were adsorbed onto the wheat straw fibers. Thermogravimetric analysis showed that a higher thermal stability of the wheat straw fiber was obtained with the modified TiO_2_ nanoparticles. The flexural, tensile, and impact properties were improved. A higher ultraviolet (UV) stability of the samples treated with modified TiO_2_ nanoparticles was exhibited by the study of the color change and loss in mechanical properties.

## 1. Introduction

Most agricultural residues are currently burned off in the field. The burning off of wheat straw and other agricultural residues is responsible not only for a series of environmental problems, but also for the sustainable development of precious resources. Therefore, using a waste biomass resource has been the main objective, scientifically and sufficiently, of many studies. Recently, many studies have been carried out on composite panels produced using various agricultural residues and plastic [1,2,3]. The most commonly investigated mechanical properties of agricultural fiber plastic composites (AFPC), such as tensile, flexural, and impact properties, are comparable to wood-plastic composites (WPC). Similar to WPC, AFPC can be extensively used for furniture, packaging, transportation, decorative materials, and so forth. However, several deficiencies, such as low strength-to-weight ratio, poor toughness, weak brittleness, and inconvenience of installation, have limited the application of AFPC [4]. Incorporating a blowing or foaming agent in the production of the AFPC could effectively avoid some of these disadvantages [5,6]. ZnO-modified azodicarbonamide (AC) has recently been widely used in research as a blowing agent owing to its compatible decomposition temperature range with the processing temperature of the plastic matrix in the AFPC [7]. The application of the AFPC as a building material necessitates that they are used outdoors and are often exposed to sunlight, which has resulted in concerns about their short service life. Of particular concern is the durability of AFPC after UV exposure. To overcome the inclination of AFPC to aging caused by UV, various approaches have been used to impart an anti-aging agent to the composite materials [8,9].

TiO_2_ is of interest for various applications owing to its numerous merits, such as low cost, nontoxicity, high photocatalytic activity, optical and electronic properties, antibacterial activity, UV protection, and environmental friendliness. When the TiO_2_ particle size is reduced to the nanoscale, a significant effect on the anti-ultraviolet aging performance, thermal stability, and mechanical properties in the polymer materials has been demonstrated [10]. Novel properties of nanocomposites can be obtained by successfully incorporating TiO_2_ nanoparticles into matrix materials. However, owing to their nanoscale size, extremely high surface area, and the ability to form highly-reactive hydroxyl and superoxide radicals, TiO_2_ nanoparticles agglomerate very easily in various media and show a poor dispersion capacity in materials, which may reduce the resultant correlation properties of the nanocomposite materials. Therefore, it is necessary to control the nanoparticle morphology by dispersion modification to make them uniformly combine with other components. Many efforts have been made to overcome this problem and to enhance the filler–matrix interaction [11]. Surface modification of TiO_2_ nanoparticles with silane coupling is an effective way to reduce its surface energy and to improve its dispersion properties. Moreover, grafting active functional groups onto the nanoparticle surface can increase the possibility of forming chemical bonds between modified nanoparticles and matrix materials. Surface-modified TiO_2_ nanoparticles have been widely applied to polymer nanocomposites. However, research to study the effects of TiO_2_ nanoparticles on foamed fiber/plastic composites is relatively rare, even though TiO_2_ nanoparticles exhibit obvious UV absorption that may cause an increase in the UV resistance of composites.

Thus, the aim of this study was to investigate the practical feasibility of using TiO_2_ nanoparticles as an additive in the manufacturing of foamed wheat straw fiber/PP composites. To achieve an appropriate dispersion of TiO_2_ nanoparticles and to yield a better compatibility between the wheat straw fiber and PP, a silane coupling agent, A171, was used for surface modification of the TiO_2_ nanoparticles. The effects of the modification with TiO_2_ nanoparticles on the mechanical properties and resistance to UV aging of the composites were evaluated.

## 2. Materials and Methods 

### 2.1. Raw Materials and Preparation

**Figure 1:**
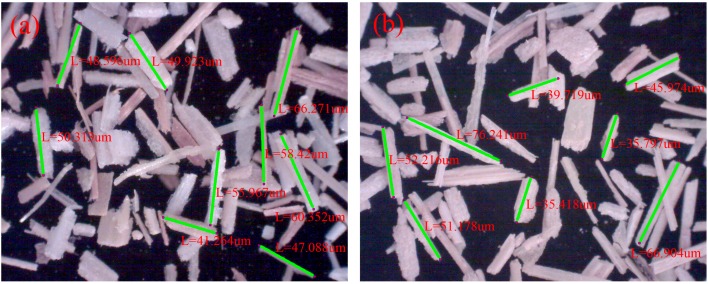
Optical microscope images of (**a**) untreated wheat straw fiber and (**b**) wheat straw fiber treated by A171 and modified TiO_2_ nanoparticles.

Wheat stalks were collected from a farmland in Weifang, China. The wheat stalks were granulated to the length of 40–80 um (Figure 1), and the materials were dried to a moisture content of approximately 3% prior to use. PP (density = 920 kg/m^3^, melt flow index (MFI) = 2.5 g/10 min) was used as a matrix. The coupling agent, ethenyltrimethoxy silane (A171, CH_2_=CHSi(OCH_3_)_3_, boiling point: 285 °C) was received from Quanxi Chemical Co., Ltd. (Nanjing, China). Azodicarbonamide (AC, decomposition temperature: 180–210 °C, gas evolution: 215–235 mL/g), as a blowing agent, was purchased from Hengrui Chemical Co., Ltd. (Jinan, China). TiO_2_ nanoparticles (purity: 99.8%, 25 nm, anatase titania), zinc oxide (ZnO), calcium carbonate (CaCO_3_), industrial paraffin, distilled water, ethanol, and acetic acid were purchased from Aladdin Reagent (Shanghai, China).

### 2.2. Modification of TiO_2_ Nanoparticles with the A171 Coupling Agent

Although the commercial and functionalized TiO_2_ nanoparticles can be obtained from many suppliers nowadays, there are some problems affecting the control quality and the stability of the coupling agent. Thus, the TiO_2_ nanoparticles used in this study were functionalized in the laboratory. The silane coupling agent, A171, was used for the modification of nano-sized TiO_2_ particles. The TiO_2_ nanoparticles were dispersed in a certain amount of ethanol, and acetic acid was added into the mixture to achieve a pH of 4. The mixture was mechanically stirred at 500 rpm at room temperature for 30 min and was then ultrasonicated for 20 min. Then, the dispersion was transferred into a 250 mL three-necked round-bottom flask fitted with a condenser and a thermometer and A171 (15% of the mass of the TiO_2_ nanoparticles) was added. The reaction for the functional groups of A171 to the TiO_2_ nanoparticle surfaces was performed at 60 °C and allowed to proceed for 1 h. The dispersion was purified of the free A171 by five cycles of centrifugation for 20 min (10,000 rpm), redispersion of the sediment in absolute ethanol, and ultrasonication for 10 min. A part of the dispersion was oven-dried at 100 °C for 24 h and was cooled in a vacuum for 1 h to obtain a modified TiO_2_ nanoparticle powder for back-up detection.

### 2.3. Modification of Wheat Straw Fiber with the A171 Coupling Agent and Modified TiO_2_ Nanoparticle Powder

Ninety-five percent ethanol was prepared by mixing pure ethanol and distilled water. A171 was added into the prepared 95% ethanol with stirring to reach a 4% concentration. Acetic acid was added into the mixture to reach pH 4 and mixed with mechanical stirring at room temperature for some hours for hydrolysis. The wheat straw fiber was placed into a high-speed mixer. Under the condition of 60 °C, 1% prepared silane solution and 0%–3% modified TiO_2_ nanoparticles were sprayed on the surface of the fiber and then the fibers were stirred for 30 min. The fiber samples were taken out and then dried at room temperature to fully evaporate the ethanol (Figure 1).

### 2.4. Blend Design and Sample Fabrication

Through a comprehensive analysis of the relevant formulations of foamed composites and nanocomposites, the design for various formulations is shown in Table 1. According to Table 1, modified wheat straw fiber, PP, AC, ZnO, CaCO_3_, and industrial paraffin were uniformly mixed in a high-speed mixer. The extrudates were prepared in the Engineering Composite Laboratory of Northeast Forestry University using a SJSH30/SJ45 twin-screw extruder (Nanjing Rubber Machinery Plant, Nanjing, China) and then granulated using a granulator. The blending temperature profile was controlled at 100, 130, 160, 165, 165, 180, and 175 °C from the feeding zone to the die. After the granulation was masticated by a two-roll mill, the composites were fabricated by prepressing for 15 min, hot-pressing under 5 MPa for 10 min, and cold-pressing for 5 min, in that order.

### 2.5. Material Characterization

#### 2.5.1. FTIR Study

The treated and untreated wheat straw fiber and TiO_2_ nanoparticles were ground and FTIR absorption data were obtained using a Nicolet 6700 FTIR spectrometer (Thermo Fisher Science, Agawam, MA, USA) with an ATR unit (Universal ATR Diamond Zn/Se) at a resolution of 4 cm^−1^ for 32 scans in the spectral range of 600–4000 cm^−1^.

#### 2.5.2. Thermogravimetric Analysis

Thermal properties of the untreated and treated wheat straw fiber samples were measured by using a TGA 309F3 thermal analyzer (TA Instruments, New Castle, DE, USA) at a heating rate of 10 °C/min from room temperature up to 600 °C under a nitrogen atmosphere.

#### 2.5.3. Accelerated Aging Test

**Figure 2:**
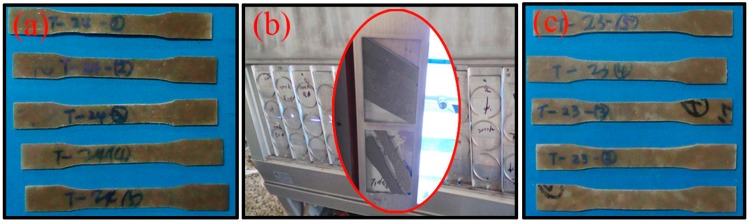
The samples of (**a**) before the UV aging test; (**b**) being UV aging tested; and (**c**) after the UV aging test.

Fifteen replicates of each of the six formulations were placed in a xenon arc-type light exposure apparatus operated according to the American Society for Testing Material (ASTM) G 154-04 standard practice, with treatment for up to 600 h (Figure 2). To understand the effect of UV exposure, the samples were removed for colorimetric analysis at an interval of 100 h. A NF333 photometer (Nippon Denshoku Co., Tokyo, Japan) was used to measure the color according to the International Commission on Illumination (CIE). The standard colorimetric parameters (brightness L*, red-green chromaticity index a*, and yellow-blue chromaticity index b*) were measured at five locations on each sample, and the color change (*ΔE*) was determined with the procedure outlined in ASTM D 2244:

(1) ΔE=(ΔL*2+Δa*2+Δb*2)12

where ΔL*, Δa*, and Δb* represent the differences between the initial and final values of L*, a*, and b*, respectively.

#### 2.5.4. Mechanical Properties

**Figure 3:**
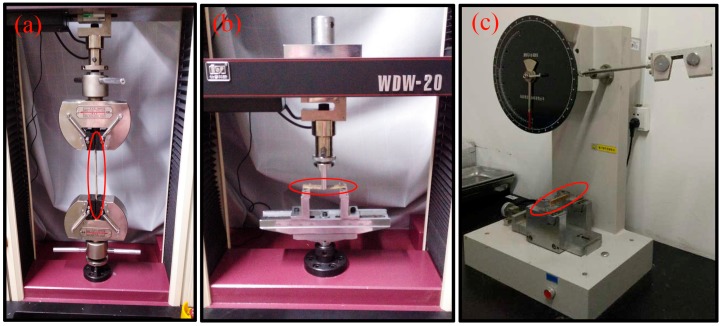
The samples’ (**a**) tensile property being tested; (**b**) the flexural property being tested; and (**c**) the impact property being tested.

Samples were oven-dried at 103 °C for 24 h before testing to ensure the same conditioning for samples before and after UV exposure. Tensile tests were conducted in accordance with ASTM D 638. The size of each sample was 165 mm length and 19 mm width. Flexural (three-point bending) strength was measured according to ASTM D 790 using a support span of 56 mm and a cross head speed of 2 mm/min. Five replicates were run for each formulation. All tests were performed on a universal testing machine (Shenzhen REGER Instrument Co., Ltd., Shenzhen, China). The impacting strengths were measured according to ASTM D 6110 on a XJ-T0G composited impacting tester (Hebei Chengde Mechanical Instrument Co., Ltd., Chengde, China). Ten replicates for each composition were tested for impact strength. The mean value of parallel specimens was used as the test result. Specimens of the mechanical properties test are shown in Figure 3.

## 3. Results and Discussion

### 3.1. FTIR Analysis

**Figure 4:**
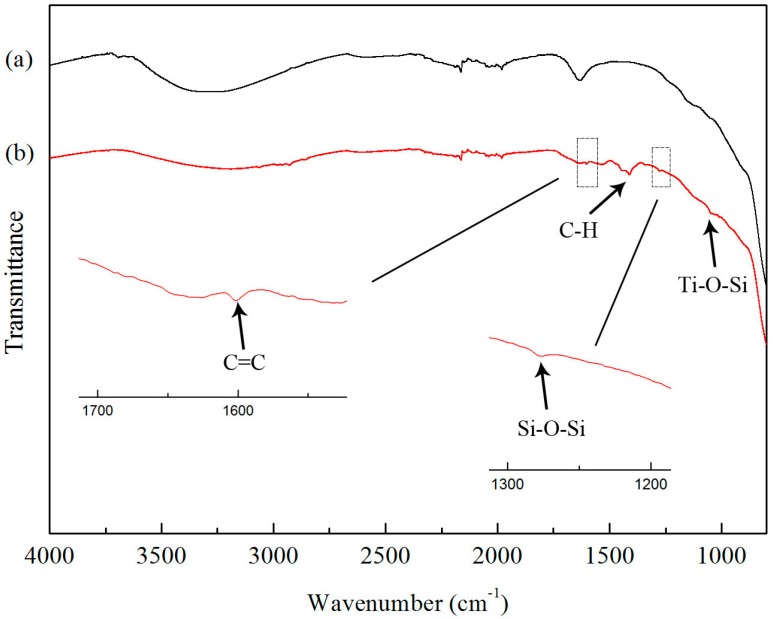
FTIR spectra of (**a**) untreated TiO_2_ and (**b**) A171-grafted TiO_2_ nanoparticles.

FTIR spectra of unmodified TiO_2_ nanoparticles and A171-modified TiO_2_ nanoparticles are presented in Figure 4. In the spectrum for the unmodified TiO_2_ (curve-4a), the stretching vibration of the absorbed water, as well as surface –OH groups, which are present in the TiO_2_ nanoparticles, were confirmed by the broad absorption band between 3400 cm^−1^ and 3200 cm^−1^ and the peak at around 1630 cm^−1^ [12]. This peaks were mainly observed because the TiO_2_ nanoparticles were easily polarized in the presence of water and produced a large quantity of hydroxyl groups on the surface. Furthermore, the electrons in the valence band of nano TiO_2_ were excited to generate electron-hole pairs, and part of the electron-hole pairs migrated to the surface, forming the hydroxyl groups. For the A171-treated TiO_2_ (curve-4b), the broad absorption peak between 3400 cm^−1^ and 3200 cm^−1^ was weakened. This indicated that a condensation reaction between the silanol groups of hydrolyzed A171 and the surface hydroxyl group of TiO_2_ nanoparticles occurred [13]. In addition, a broad band at around 1276.16 cm^−1^ corresponding to a Si–O–Si bond was observed, which confirmed a condensation reaction between silanol groups hydrolyzed by A171 [11]. Compared with pure TiO_2_ nanoparticles, the characteristic bands of 2958.43 cm^−1^ and 1620 cm^−1^ were attributed to the –CH_3_ stretching and C=C stretching, respectively, demonstrating that the functional groups in A171 were grafted onto the TiO_2_ nanoparticle surface. Zhao et al. [11] showed that according to the IR spectra of modified nano TiO_2_, the peak at 940 cm^−1^–910 cm^−1^ was assigned to the stretching vibration band of Ti–O–Si. Chen and Yakovlev [14] also reported that the Si–O–Ti bonds confirmed the bonding of organosilane functional groups on the particle surface. Since the residual (non-reacted) and physically-adsorbed A171 molecules were removed by extraction in ethanol and centrifugal separation, the peaks mentioned showed that the chemical bonding of A171 onto the nanoparticle surfaces was realized through Ti–O–Si bonds, which could improve the dispersion of TiO_2_ nanoparticles, mechanical properties, and UV aging resistance of composites containing TiO_2_ nanoparticles [15].

**Figure 5:**
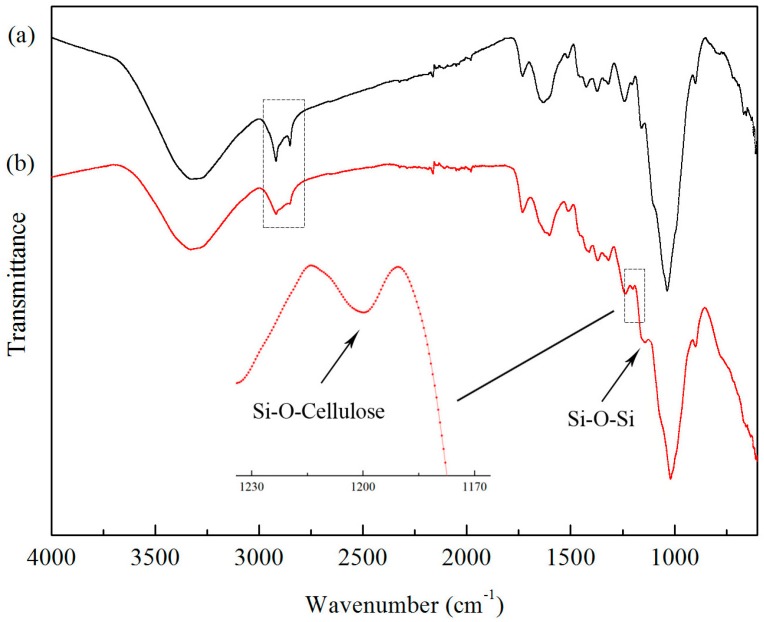
FTIR spectra of (**a**) untreated wheat straw fiber and (**b**) wheat straw fiber treated by A171 and modified TiO_2_ nanoparticles.

Figure 5 shows the FTIR spectra of pure wheat straw fiber and modified wheat straw fiber. The pure wheat straw fiber was characterized by the absorption bands (shown in curve-5a) appearing at between 3598.7 cm^−1^ and 3257.18 cm^−1^, which were associated with the stretching vibration band of intramolecular –OH of cellulose, hemicellulose, polysaccharide, and monosaccharide in the wheat straw fiber. The C–H stretching vibrations of –CH_3_, as well as –CH_2_, which were present in the cellulose, were confirmed by the peaks at 2853.17 cm^−1^ and 2918.25 cm^−1^, respectively. The cellulose was characterized by a high-intensity peak at 1034.14 cm^−1^ for –OH bending and shoulder peaks appeared at around 1158.53 cm^−1^ and 897.70 cm^−1^ for C–O–C stretching. The absorption peak at 896.25 cm^−1^ was ascribed to the intramolecular hydrogen bonding [16]. After surface modification by organosilane, as presented in spectra curve-5b, the characteristic peaks for wheat straw fiber and organosilane were observed. The broad absorption peaks at 2853.17 cm^−1^ and 2918.25 cm^−1^ were significantly weakened, indicating the quantity of C–H presented in cellulose was changed by the silane coupling agent. The peak that appeared at 1134.42 cm^−1^ for the Si–O–Si bond was due to the condensation reaction of silanol groups hydrolyzed by A171. The absorption peak at 1200.47 cm^−1^ was ascribed to the Si–O–cellulose bond, indicating that a strong polycondensation grafting reaction between silanol groups and wheat straw fiber occurred [12]. No characteristic peaks for Ti–O–cellulose or hydrogen bonds formed between the TiO_2_ surface –OH and the wheat straw fiber surface –OH were observed, showing that the chemical reaction between the wheat straw fiber and the modified TiO_2_ nanoparticles did not occur. This proved that the main bonding mechanism of the modified TiO_2_ nanoparticles and wheat straw fiber was physical sorption. However, Sun et al. showed that TiO_2_ was chemically bonded to the wood surface through the hydrogen groups during the hydrothermal process [17]. This may arise from the different reaction conditions.

### 3.2. Morphology and Dispersibility of Surface-Modified TiO_2_ Nanoparticles

**Figure 6:**
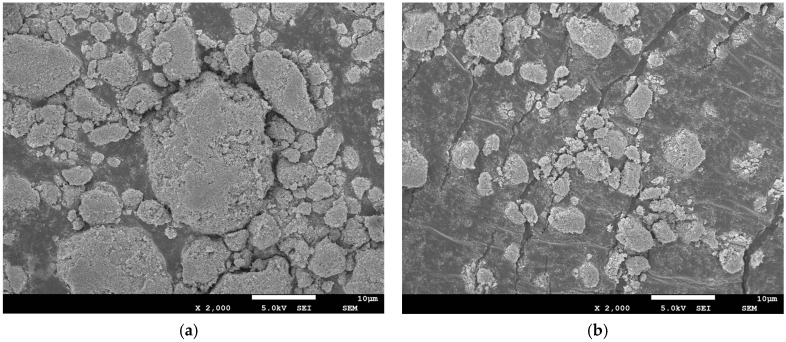
SEM images of (**a**) untreated TiO_2_ and (**b**) A171-grafted TiO_2_ nanoparticles.

SEM analysis was conducted to examine the morphology and dispersibility of surface-modified TiO_2_ nanoparticles. Typical surface SEM images of pure TiO_2_ nanoparticles and surface-modified TiO_2_ nanoparticles are illustrated in Figure 6. As can be seen from Figure 6a, the spherical shape, as well as agglomerates, comprised the unmodified TiO_2_ nanoparticles, indicating a poor dispersion in the control group. From Figure 6b, an improved dispersion was observed in the surface-modified TiO_2_ nanoparticles, showing that the surface modification prevented the aggregation of TiO_2_ nanoparticles to some extent. Certainly, there were still a small amount of agglomerates in the surface-modified TiO_2_ nanoparticles, which might have an impact on some properties.

### 3.3. Thermogravimetric Analysis

**Figure 7:**
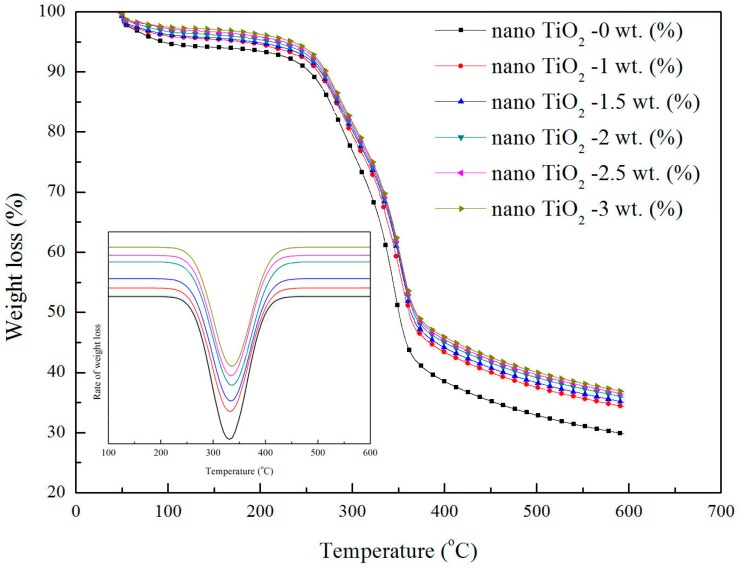
TGA curves of wheat straw fiber with different surface-modified TiO_2_ nanoparticle contents.

To estimate the optimum amount of modified TiO_2_ nanoparticles required to enhance the properties of the wheat straw fiber, the wheat straw fiber treated by various percentages of modified TiO_2_ nanoparticles were analyzed by the TGA technique. The TGA curves of the wheat straw fiber with different percentages of modified TiO_2_ nanoparticles is shown in Figure 7. Three main mass loss regions were observed. In the thermograms obtained from the TiO_2_ nanoparticle-treated wheat straw fibers, all of the samples showed an initial mass loss in the region of 50–100 °C, which was attributed to the evaporation of water and residual solvents. It was evident that the mass loss was greater in the untreated wheat straw fibers compared with the treated fibers in the first stage, which arose from the untreated wheat straw fibers not being completely dry. The weight loss at this stage depends mainly on the initial moisture content of the fiber, therefore, the thermal properties should be analyzed from the second stage of thermal degradation. The second main degradation region was located between 200 °C and 350 °C, and the third stage mass loss occurred above 350 °C. The second degradation region was attributed to the cellulose and also to the degradation of lignin and other low molecular weight substances, which agreed with the results from the study of Huang et al. [18]. As can be seen, the treated wheat straw fiber had a higher decomposition temperature than the untreated wheat straw fiber, indicating the thermal stability of the fiber was enhanced by adding the modified TiO_2_ nanoparticles. The degradation temperature of the treated wheat straw fiber was slightly increased with the TiO_2_ nanoparticle content. Compared with the untreated wheat straw fiber, a reduced rate of the degradation of the wheat straw fiber became evident with the gradual increase in the additional amount of modified TiO_2_ nanoparticles. This further demonstrated that the modified TiO_2_ nanoparticles played a role on the thermal stability of the wheat straw fiber. Another slight weight loss after 350 °C may be attributed to the water formed from the condensation of the hydroxyl group on the particle surface [15]. In the thermal degradation process, 61% and 77% weight loss were observed for the untreated and treated wheat straw fiber, respectively. The percentages of modified TiO_2_ particles had no obvious effect on the thermal properties of wheat straw fiber within the range selected for this experiment. For higher 1 wt.% content, the effect of TiO_2_ nanoparticles on the thermal properties of wheat straw fiber was small. Hence, it was concluded that the thermal stability of the obtained wheat straw fiber was slightly improved with the dispersion of modified TiO_2_ nanoparticles.

### 3.4. Color Change during UV Aging

**Figure 8:**
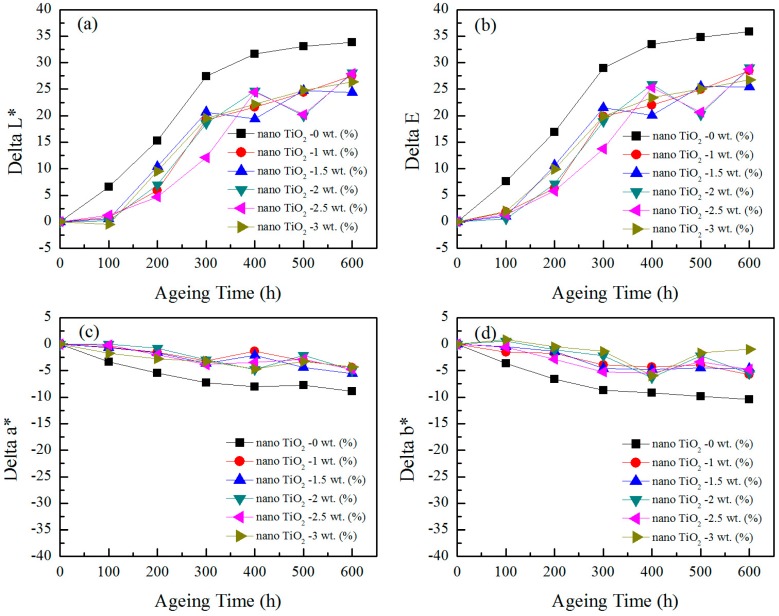
The change in color of foamed wheat straw fiber/PP composites with different modified TiO_2_ nanoparticle content after UV weathering.

The values of ΔL*, Δa*, Δb*, and *ΔE* at different exposure times for UV-aged composites made from six different formulations are shown in Figure 8. From the figure, it was obvious that the brightness factor ΔL* and the calculated total *ΔE* increased for all the samples with an increasing exposure, which was indicative of color fading of the UV-aged composite samples. Although all of the samples experienced an increase in the value of ΔL* and *ΔE*, the increment of the value was not proportional to the increase of exposure. Within the 300–400 h block, there was an apparent growth rate, and then the growth rate gradually decreased. These findings were consistent with the study of Fabiyi [19]. The samples without TiO_2_ nanoparticles exhibited larger ΔL* and *ΔE* values than samples with TiO_2_ nanoparticles, indicating the enhanced UV aging-resistant performances for TiO_2_ nanoparticle-added materials, which arose from the UV shielding ability of TiO_2_ nanoparticles. Du et al. [9] and Hou et al. [20] reported that the color fading of wood-plastic composites could be attributed to the photodegradation nature of the fiber. Therefore, nano TiO_2_ could inhibit color fading by absorbing some of the UV radiation, which resulted in less UV radiation being available to degrade the color of the samples. Furthermore, surface modification of TiO_2_ nanoparticles by silane has been found to be effective in improving its dispersion. The TiO_2_ particles with better dispersion enhanced the UV protection and improved the weathering performance [21]. As for the effect of the TiO_2_ nanoparticle content on the color fading of composites, no significant differences were recorded among the TiO_2_ nanoparticle-incorporated formulations, showing that the TiO_2_ nanoparticle content level had little effect on the anti-ultraviolet aging performance within the selected range of this experiment. This may be related to the presence of a small amount of agglomerates after the surface modification of the TiO_2_ nanoparticles. When comparing the effect of TiO_2_ nanoparticles on the values of Δa* and Δb*, it can be seen that these values decreased for all samples. Compared with ΔL*, the absolute values of Δa* and Δb* were relatively small, showing the Δa* and Δb* were not the main influencing factors of *ΔE*. Since the trend of *ΔE* followed that of ΔL*, ΔL* was the most important parameter influencing the total *ΔE*. A similar observation was reported by Hou [20]. This color fading pattern, therefore, revealed that composite formulations with moderated weathering performance could be achieved by incorporating TiO_2_ nanoparticles. Du et al. [9] also reported that the UV stability of wood–polymer composites was increased by incorporating ultraviolet absorbers.

### 3.5. Mechanical Properties Study

**Figure 9:**
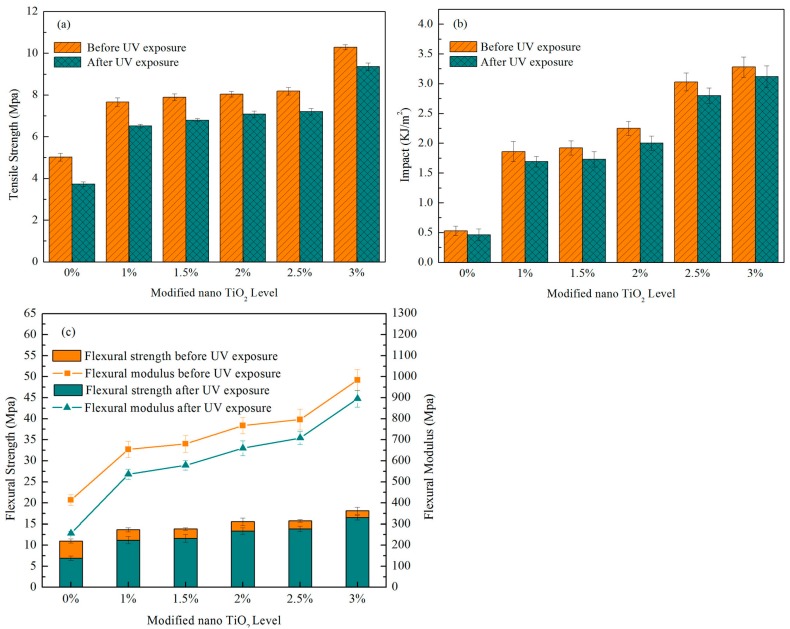
The change in mechanical properties of wheat straw fiber/PP composites with different modified TiO_2_ nanoparticle content after UV weathering.

The results of flexural, tensile, and impact properties of foamed wheat straw fiber/PP composites with different formulations before and after UV weathering are summarized in Figure 9. It was observed that the composites containing modified TiO_2_ nanoparticles exhibited higher mechanical properties than those of composites without fillers. This might be a result of the nanoscale particles having a high aspect ratio, which offers an active surface area, and could play a role on the adsorption of hydroxyl groups on the surface of the fiber and chemical bonds in PP; hence, the mechanical properties were reinforced. An improvement in the mechanical properties arising from the incorporation of nanoparticles into the composites has already been reported [12,22]. Xiao [23] showed that the mechanical properties for wood fiber/PP composite improved owing to the incorporation of Al_2_O_3_ nanoparticles. The trends of the mechanical properties were similar to each other, which showed a rising tendency along with the increasing concentration of modified TiO_2_ nanoparticles, except for a small increase at a low level of modified TiO_2_ nanoparticle loading (1%–2.5%). As clearly seen from Figure 9, the control samples showed lower mean mechanical values of 11.02 MPa, 5.02 MPa, and 0.53 KJ/m^2^ for flexural, tensile, and impact properties, respectively. When the modified TiO_2_ nanoparticle content was increased to 3%, the mechanical values correspondingly increased to 18.13 MPa, 10.29 MPa, and 3.28 KJ/m^2^. In general, the addition of modified nano TiO_2_ affected the mechanical properties. However, in the range of the modified TiO_2_ nanoparticle additions selected in this experiment, no optimal values were obtained. The effect of higher modified TiO_2_ nanoparticle contents (above 3 wt.%) on the mechanical properties of epoxy nanocomposites were examined in previous studies [24]. The influence of a high modified TiO_2_ nanoparticle content on the mechanical properties of the foamed wheat straw fiber/PP composites remains to be studied.

From Figure 9 and Table 2, the flexural, tensile, and impact properties were reduced after UV treatment. The reduction in the mechanical properties was greater in the control samples. The modified TiO_2_ nanoparticle-added formulations exhibited less of a reduction in all flexural, tensile, and impact properties. This might be a result of the modified TiO_2_ nanoparticles inhibiting the rate of photodegradation. Related research has shown that ultraviolet light absorbed by TiO_2_ nanoparticles is converted into heat, and the remaining UV energy is not sufficient to destroy the composites. Therefore, the resistance of foamed wheat straw fiber/PP composites to UV instability could be significantly improved with the addition of modified TiO_2_ nanoparticles. However, the loss of mechanical properties for the composites incorporating any level of modified TiO_2_ nanoparticles was unremarkable for the studied period of UV irradiation. These findings were consistent with the above analysis, where the range of modified TiO_2_ nanoparticle additions selected in this experiment was limited. Moreover, the effect of a small amount of agglomerates formed in the surface modified TiO_2_ nanoparticles could not be ignored. A study for a lower reduction in the mechanical properties and better UV resistance of these composites by adding more TiO_2_ nanoparticles is still needed. As with the composites without UV aging, the mechanical properties were consistently high for all composites containing modified TiO_2_ nanoparticles compared with the control specimens. This may arise from the interfacial interaction between the PP matrix, modified TiO_2_ nanoparticles, and fibers, which is powerful enough to delay aging resulting from exposure to UV irradiation. Furthermore, the UV aging resulted in a relatively higher reduction of flexural properties in all composites with different formulations when compared with the tensile and impact properties, indicating the ultraviolet radiation had a greater influence on the flexural properties. This was mainly because the PP was oxidized under ultraviolet light and the aerobic environment and, as a result, the PP chains were fractured. The chain scission of the PP increased the microcracks on the composite surfaces, which led to a decrease in the flexural properties and hindered the stress transfer from the filler to the matrix, causing a decrease in the composite strength. In addition, since flexural strength depends on the tensile strength of the composite, the decreased flexural strength can be attributed to the decreased tensile modulus.

## 4. Conclusions

TiO_2_ nanoparticles modified by A171 were used as the active fillers for foamed wheat straw fiber/PP composites imparting an anti-UV performance to the materials. Surface modification of the TiO_2_ nanoparticles with a silane coupling agent and an interaction between the wheat straw fiber and the silane coupling agent were successfully achieved. Thermogravimetric analysis showed that a higher thermal stability was achieved for the wheat straw fiber treated with modified TiO_2_ nanoparticles. Improvements in the flexural, tensile, and impact properties were obtained, which could be attributed to the active surface area of the modified TiO_2_ nanoparticles. The samples treated with modified TiO_2_ nanoparticles were shown to exhibit a higher UV stability than that of untreated nanoparticles by studying the color change and loss in mechanical properties. Basic research on the application of TiO_2_ nanoparticles incorporated into foamed wheat straw fiber/PP composites was conducted and will aid in future research in this field.

## Figures and Tables

**Table 1:** Formulations of various samples for the preparation of the foamed wheat straw fiber/PP composites.

E	Modified TiO_2_ Nanoparticles (wt.%)	Silane Coupling Agent (wt.%)	Wheat Straw Fiber (wt.%)	PP (wt.%)	AC (wt.%)	ZnO (wt.%)	CaCO_3_ (wt.%)	Paraffin (wt.%)
A	0	1	29	62.5	2	0.5	4	1
B	1	1	29	61.5	2	0.5	4	1
C	1.5	1	29	61	2	0.5	4	1
D	2	1	29	60.5	2	0.5	4	1
E	2.5	1	29	60	2	0.5	4	1
F	3	1	29	59.5	2	0.5	4	1

**Table 2:** The change and reduction ratio in mechanical properties of wheat straw fiber/PP composites with different modified TiO_2_ nanoparticle content after UV weathering.

Modified Nano TiO_2_ Level (wt.%)			0	1	1.5	2	2.5	3
Flexural properties	Before UV exposure	Flexural strength (MPa)	11.02	13.66	13.85	15.56	15.74	18.13
		Flexural modulus (MPa)	413.59	653.62	680.33	767.42	795.99	984.05
	After UV exposure	Flexural strength (MPa)	6.89	11.2	11.63	13.38	13.85	16.5
		Flexural strength loss (%)	37.48	18.01	16.03	14.01	12.01	8.99
		Flexural modulus (MPa)	256.06	535.97	578.28	659.98	708.43	895.49
		Flexural modulus loss (%)	38.09	18.00	15.00	14.00	11.00	9.00
Tensile properties	Before UV exposure	Tensile strength (MPa)	5.02	7.66	7.89	8.04	8.18	10.29
	After UV exposure	Tensile strength (MPa)	3.72	6.51	6.79	7.08	7.2	9.36
		Tensile strength loss (%)	25.90	15.01	13.94	11.94	11.98	9.04
Impact properties	Before UV exposure	Impact (KJ/m^2^)	0.53	1.86	1.92	2.25	3.03	3.28
	After UV exposure	Impact (KJ/m^2^)	0.46	1.69	1.73	2	2.8	3.12
		Impact loss (%)	13.21	9.14	9.90	11.11	7.59	4.88
